# Adipose-Derived Stem Cells Protect Ischemia-Reperfusion and Partial Hepatectomy by Attenuating Endoplasmic Reticulum Stress

**DOI:** 10.3389/fcell.2020.00177

**Published:** 2020-03-20

**Authors:** Zhihui Jiao, Xiaoning Liu, Yajun Ma, Yansong Ge, Qianzhen Zhang, Boyang Liu, Hongbin Wang

**Affiliations:** ^1^College of Veterinary Medicine, Northeast Agricultural University, Harbin, China; ^2^Heilongjiang Key Laboratory for Laboratory Animals and Comparative Medicine, Northeast Agricultural University, Harbin, China; ^3^College of Animal Science and Technology, Heilongjiang Bayi Agricultural University, Daqing, China

**Keywords:** ADSCs, ERS, ischemia-reperfusion, hepatectomy, laparoscopy

## Abstract

Ischemia-reperfusion (IR) is an inevitable complication of liver surgery. Recent studies indicate a critical role of endoplasmic reticulum stress (ERS) in hepatic IR. Mesenchymal stem cells (MSCs) have proven to be an effective tool for tissue regeneration and treatment of various diseases, including that of the liver. However, the mechanisms underlying the therapeutic effects of stem cells on hepatic IR injury (IRI) are still poorly understood, especially in the context of ERS. In this study, we established a porcine model of hepatic IRI and partial hepatectomy, and transplanted the animals with adipose-derived mesenchymal stem cells (ADSCs) isolated from miniature pigs. ADSCs not only alleviated the pathological changes in the liver parenchyma following IRI, but also protected the resident hepatocytes from damage. Mechanistically, the ADSCs significantly downregulated ERS-related proteins, including GRP78, p-eIF2α, ATF6 and XBP1s, as well as the proteins involved in ERS-induced apoptosis like p-JNK, ATF4 and CHOP. Taken together, ADSCs can alleviate hepatic IRI by inhibiting ERS and its downstream apoptotic pathways in the hepatocytes, indicating its therapeutic potential in liver diseases.

## Introduction

The endoplasmic reticulum (ER) is the site of protein synthesis, post-translational modifications, folding and trafficking. In addition, it also regulates the cellular stress response and calcium levels, apart from synthesizing cholesterol, steroids and other lipids. ER stress (ERS) is characterized by protein misfolding, accumulation of the aberrant proteins and Ca^2+^ imbalance, which triggers the unfolded protein response (UPR) (Kozutsumi et al., 1988) and the apoptotic pathway. The UPR in mammalian cells is mediated by the PKR-like ER kinase (PERK), activating transcription factor 6 (ATF6) and inositol-requiring enzyme 1 (IRE-1) pathways, which can also trigger apoptosis if severe or sustained ERS damages ER function. The primary effect of UPR is to inhibit protein translation by inducing ER-resident chaperones like glucose-regulated protein 78 (GRP78). Although UPR can alleviate the ERS and protect cells from apoptosis (Kim et al., 2008), prolonged UPR can trigger cell death and inflammatory responses (Kim et al., 2008; Lenna and Trojanowska, 2012).

Recent studies have demonstrated a crucial role of ERS in hepatic ischemia-reperfusion (IR), which is inevitable in complex liver surgery and causes severe hepatocyte damage. Reduction of IR injury (IRI) is a major clinical challenge at present (Zhang et al., 2018, 2019; Ge et al., 2019). IR is accompanied by hypoxia, glucose/nutrient depletion, ATP depletion, excessive free radical production and Ca^2+^ imbalance, all of which can cause ER dysfunction and trigger ERS. Several studies have implicated ERS in the pathophysiology of liver IRI (Liu et al., 2012; Li et al., 2018; Obert et al., 2018), wherein it aggravates the condition by inducing apoptosis (Liu et al., 2012; Folch-Puy et al., 2016; Li et al., 2018; Xie et al., 2018). Therefore, the ERS response is a potential therapeutic target for alleviating liver IRI.

Adipose-derived mesenchymal stem cells (ADSCs) are ideal seed cells for cell-based regenerative therapies are widely used in tissue engineering (Iaquinta et al., 2019). Studies in animal models have shown that mesenchymal stem cells (MSCs) can alleviate intervertebral disc degeneration (Liao et al., 2019), acute myocarditis (Zhang et al., 2017b), obesity-associated kidney injury (Li et al., 2019), systemic lupus erythematosus (Guo et al., 2015) etc. by inhibiting ERS and apoptosis in the injured cells. In addition, MSCs can also protect the kidney from IRI by reducing ERS (Wang et al., 2019). To the best of our knowledge, no study so far has examined the therapeutic effects of ADSCs on hepatic IRI. To this end, we established a model of hepatic ischemia-reperfusion and partial hepatectomy in miniature pigs, and transplanted the animals with ADSCs. These stem cells visibly attenuated the pathological and cellular damage in the liver by inhibiting ERS and ERS-induced apoptosis. Our findings provide new insights into the therapeutic potential of ADSCs in liver diseases.

## Materials and Methods

### Animals and Groupings

Eighteen 4–6 months old Bama miniature pigs weighing 20–25 kg (half of them male and half female) were provided by the College of College of Veterinary Medicine (Harbin, China) and randomly divided into the sham-operated (Sham), IRI and ADSCs-transplanted (ADSCs) groups (six animals per group). The animals were housed in a temperature and humidity-controlled environment under a 12 h light/dark cycle with *ad libitum* access to piglet food (Shenzhen Jinxinnong Feed, China) and water. The experiments were approved by the Animal Care and Use Committee of the Northeast Agricultural University (SQ-2019-0209), and conducted in accordance with the guidelines for the care and use of experimental animals established by the Ministry of Science and Technology of the People’s Republic of China.

### Isolation and Characterization of ADSCs

Adipose-derived mesenchymal stem cells were harvested from the abdomen of the miniature pigs. Briefly, the adipose tissue was removed and digested with 0.01% collagenase I (Biosharp, China), and re-suspended in L-DMEM supplemented with 10% FBS (Clark, United States), 2 mM L-glutamine, 1 μg/ml penicillin and 100 μg/ml streptomycin (all obtained from Solarbio, China). The single cells were seeded into 25 cm^2^ cell culture flasks (Corning, United States) and cultured at 37°C under 5% CO_2_ (Galaxy 170 S, Eppendorf, Germany). The ADSCs from the third to fifth passages were incubated with anti-porcine FITC-conjugated antibodies against CD29, CD34, CD44, and CD105 (1:1000, Abcam, United States). After washing twice with PBS, the stained cells were acquired in a Coulter flow cytometer and analyzed using the FACSD software (BD, United States). Adipogenic, osteogenic and hepatic differentiation was analyzed by culturing the cells in the respective differentiation media (Cyagen Biosciences, United States) according to the manufacturers’ instructions.

### Surgical Procedure

**FIGURE 1:**
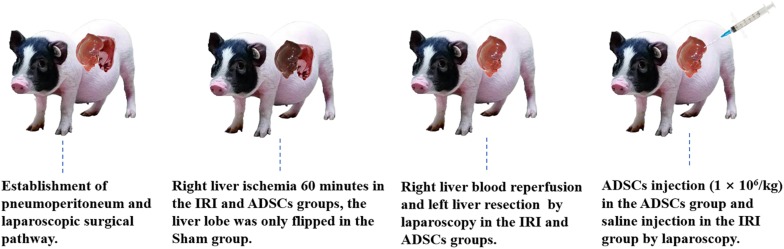
The surgical procedure.

After overnight fasting, the animals were anesthetized with 2.5–4% isoflurane. CO_2_ was injected into the abdominal cavity via a veress needle, and the pneumoperitoneum pressure was maintained at 10 mm Hg. Left hepatectomy and right hepatic ischemia for 60 min was performed in the IRI and ADSCs groups by laparoscopy as described previously (Zhang et al., 2014), while the liver lobe was only flipped in the Sham group. The animals in the ADSCs group were injected ADSCs through the liver parenchyma by laparoscopic instruments immediately after hemi-hepatectomy (10^6^ cells/kg). ADSCs at P3-P5 were resuspended in saline for allogeneic transplantation. The surgical procedure was shown in Figure 1. Tolfedine 4% (Vetoquinol S.A, France) was administered to all animals after the operation. The abdominal incision suture was removed 7 days after the operation. All surgeries are performed skillfully by veterinarians trained in laparoscopic surgery in the same environment. The liver tissues were collected from the same site in the right lobe both preoperatively (after the anesthesia) and 1 day after the operation, and stored at −80°C.

### Histological Analysis

The resected tissues were fixed with 10% buffered formalin, embedded in paraffin, and cut into 4 μm-thick sections. The tissue sections were stained with hematoxylin and eosin (H&E) as per standard protocols, sealed with neutral balsam, and observed by light microscopy. Histopathological score was according to the Suzuki classification (Suzuki et al., 1993). Refer sinusoidal congestion, vacuolization of hepatocyte cytoplasm and parenchymal necrosis for scoring criteria.

### Transmission Electron Microscopy

Fresh liver specimens were immediately fixed in 2.5% glutaraldehyde for 2 h at 4°C, washed with PBS and post-fixed in 1% osmic acid for 2 h. The fixed samples were dehydrated in graded alcohol, embedded in resin, cut into ultrathin sections and then stained with uranyl acetate and lead citrate. The stained sections were observed under a transmission electron microscope (TEM; H-7650, Hitachi, Japan).

### Immunohistochemistry

The tissue sections were incubated with 3% H_2_O_2_ for 10 min in the dark to quench endogenous peroxidases, followed by antigen retrieval using sodium citrate under high pressure steam. After cooling to room temperature, the sections were washed with PBS, blocked with bovine serum albumin (BSA) and incubated overnight with the primary antibody (1:200, GRP78, WL0781, Wanleibio, Shenyang China) at 4°C. The sections were then washed with PBS and incubated with the streptavidin-labeled HRP (PV-6001, ZSGB, Beijing China) at room temperature for 30 min, followed by incubation with DAB solution for 3 min. After counterstaining with hematoxylin, the sections were analyzed using the Image-Pro Plus 6.0 software (Media Cybernetics, Silver Spring, MD, United States). For each section, five randomly selected views (×400) were analyzed.

### Real-Time Quantitative PCR

Total RNA was extracted from liver samples using the TRIzol reagent (Invitrogen, China) according to the standard protocol. The quality and concentration of the RNA were assessed by NanoDrop^TM^ One/One (Thermo Fisher Scientific, United States). The cDNA was synthesized using Prime Script^TM^ RT reagent Kit (Takara, Japan), followed by real-time qPCR in a Light Cycler 480 (Roche Applied Science, Penzberg, Germany) according to the manufacturer’s instructions. The reaction parameters were as follows: pre-denaturation at 95°C for 30 s, followed by 40 cycles of denaturation at 95°C for 5 s, annealing and elongation at 60°C for 1 min. The relative abundance of the mRNAs was calculated according to the 2^–ΔΔCt^ method (Livak and Schmittgen, 2001). The primers were synthesized by Sangon Biotech (Shanghai, China) and are listed in Table 1. Three technical replicates were tested for each sample, and the expression of each gene was analyzed in three biological replicates.

**TABLE 1:** Gene-specific primers used in the qPCR.

Gen	Accession number	Primer sequences(5′–3′)
GRP78	XM-001927795.4	Forward: TCGGCGATGCAGCCAAGAAC Reverse: CGGGTCATTCCATGTCCGGC
ATF6	XM-013996840.1	Forward: ACCCTGTTTGCTGAACTTGG Reverse: CAAGGCACCAAATCCAAATC
ATF4	NM-001123078.1	Forward: TCAGTGCCTCAGACAACAGC Reverse: GCATGGTTTCCAGGTCATCT
XBP1s	NM_001271738.1	Forward: TTGTCACCCCTCCAGAACATC Reverse: ATGCCCAAGAGGATATCAGACTCA
CHOP	NM-001144845.1	Forward: AAGACCCAGGAAACGGAAAC Reverse: GAGCCGTTCGTTCTCTTCAG
β-actin	XM-021086047.1	Forward: TCTGGCACCACACCTTCT Reverse: TGATCTGGGTCATCTTCTCAC

### Western Blotting

Liver tissues were homogenized using Tissue Protein Extraction Reagent supplemented with 1 mM PMSF (Beyotime, Shanghai, China) and phosphatase inhibitor (MCE, Monmouth Junction, United States) in an automated fast sample grinder (Jingxin, Shanghai, China). The protein concentration was determined with a Bicinchoninic Acid (BCA) Protein Assay Kit (Beyotime, China). Equal amounts of protein per sample were separated by sodium dodecyl sulfate-polyacrylamide gel electrophoresis (SDS-PAGE), and the bands were transferred to nitrocellulose (NC) membranes using a transfer buffer. The membranes were blocked with 5% BSA for 2 h at room temperature. After washing thrice with Tris-buffered saline (TBS) containing Tween 20 (TBST) for 30 min, the membranes were incubated overnight with the primary antibodies against GRP78, ATF4, ATF6, CHOP, XBP1s, β-actin (Wanleibio, Shenyang, China), JNK, p-JNK (ImmunoWay, Plano, United States), eIF2α and p-eIF2α (Abcam, Cambridge, United Kingdom) at 4°C. The membranes were washed again with TBST for 30 min and then incubated with horseradish peroxidase (HRP)-conjugated anti-species secondary antibody (1:5000, Wanleibio, Shenyang, China) for 2 h. The bands were developed using Western Bright ECL reagent (Advansta, United States), imaged using a Tanon 5200 Imaging System (Tanon Science & Technology Co., Ltd., Shanghai, China), and quantified using ImageJ software.

### Statistical Analysis

Data were expressed as mean ± standard deviation (SD) and analyzed using GraphPad Prism 7.0 (Graph Pad Software, United States). One-way analysis of variance (ANOVA) was used to compare between groups, and *P* < 0.05 was considered statistically significant.

## Results

### Isolation and Characterization of ADSCs

**FIGURE 2:**
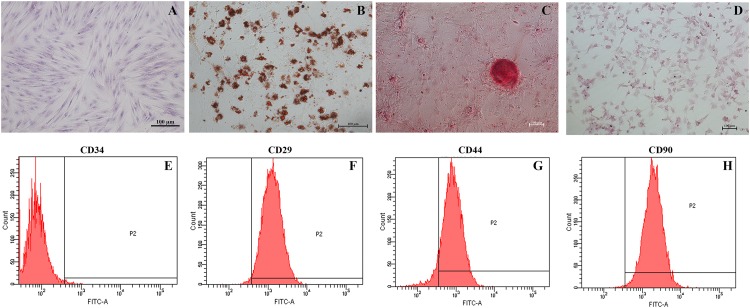
Identification of ADSCs. **(A)** Wright’s staining of passage three spindle-shaped ADSCs (magnification 200×). **(B)** Oil red O-stained lipid droplets in adipogenic cells (magnification 200×). **(C)** Alizarin Red-stained calcium crystals in osteogenic cells (magnification 100×). **(D)** PAS-stained glycogen masses in hepatic cells (magnification 100×). **(E–H)** Flow cytometry analysis of ADSCs indicating CD34^–^ CD29^+^ CD44^+^ CD90^+^ immunophenotype.

Adipose-derived mesenchymal stem cells are identified on the basis of morphology, immunophenotype and differentiation potential. The cells harvested from porcine abdomen adhered to the plastic surface, and acquired the spindle morphology of fibroblasts by the third passage (Figure 2A). The ADSCs were subjected to adipogenic, osteogenic and hepatic differentiation in suitable media. Oil red O staining showed the formation of lipid droplets following a 14-day culture in adipogenic media (Figure 2B). The ADSCs cultured in osteogenic media for 21 days showed the presence of alizarin red-stained calcium crystals (Figure 2C), and PAS-positive glycogen granules were seen after hepatic differentiation (Figure 2D) for 21 days. Finally, the ADSCs were negative for CD34 (1.1%), and expressed CD29 (98.9%), CD44 (92.4%) and CD90 (99.6%) (Figures 2E–H).

### ADSC Transplantation Alleviated Hepatic IRI

**FIGURE 3:**
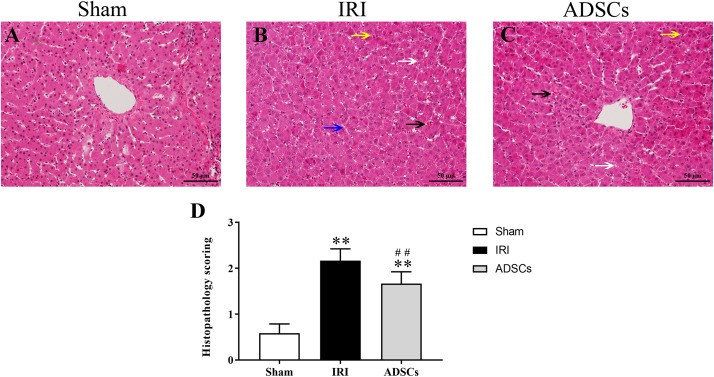
Histopathological changes and score in the liver post-IR. HE-stained liver tissues in the **(A)** sham-operated, **(B)** IRI, and **(C)** ADSCs groups. Black arrows indicate hemorrhage, white arrows indicate hepatocyte vacuolar degeneration, blue arrow indicates hepatic cord structural disorder, and yellow arrows indicate inflammatory cell infiltration (Magnification × 200). **(D)** Histopathology score. ^∗∗^*P* < 0.01, vs. sham group; ##*P* < 0.01, vs. IRI group.

**FIGURE 4:**
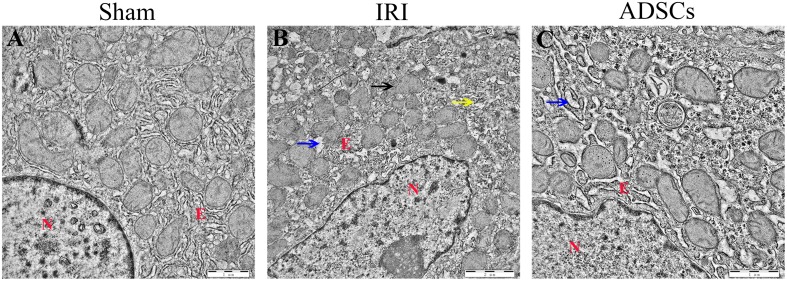
Ultrastructural changes in the liver post-IR. TEM micrographs of the liver in **(A)** sham-operated, **(B)** IRI, and **(C)** ADSCs groups. The blue arrow indicates ER swelling, the yellow arrow indicates the disordered structure of ER, and the black arrow indicates the mitochondrial swelling and the disappearance of mitochondrial ridge. N, nuclei; E, endoplasmic reticulum.

The liver tissues of the sham-operated animals showed no pathological changes in the hepatocytes and the parenchymal structure (Figure 3A). In contrast, the symptoms of IRI such as focal hepatic necrosis, severe vacuolar degeneration, hepatocyte swelling, hepatocyte cord arrangement disorder and inflammatory cell infiltration appeared within a day after the partial hepatectomy (Figure 3B). Transplantation of the ADSCs significantly alleviated IRI, as indicated by minor lesions in the parenchyma, slightly swollen liver cells, and regularly arranged hepatocyte cords. In addition, the degree of vacuolar degeneration, hemorrhage and necrotic foci were also significantly reduced in the ADSCs-treated animals (Figure 3C). The histopathology scoring was shown in Figure 3D. The liver histological damage score in the IRI group was significantly higher than that in the sham and ADSCs groups (*P* < 0.01). After stem cells transplantation, the liver injury score of ADSCs group was significantly lower than that of IRI group (*P* < 0.01). The ultrastructural changes in the hepatocytes were also analyzed in all groups by TEM. As shown in Figure 4A, the hepatocytes of the sham-operated animals had normal morphology with intact cell and nuclear membranes. IRI resulted in significant swelling and uneven ridges of the hepatocyte mitochondria, along with structural disruption and expansion of the ER (Figure 4B). ADSC transplantation alleviated the damage in both organelles (Figure 4C). Taken together, ADSCs protects the liver from IRI and its therapeutic effects are likely mediated via neutralization of ERS.

### ADSCs Blocked ERS and ERS-Induced Apoptosis in the Hepatocytes

**FIGURE 5:**
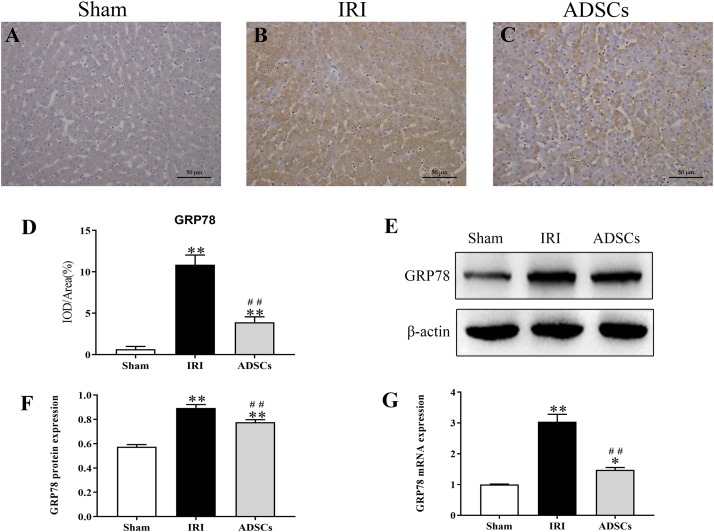
Effect of ADSCs transplantation on GRP78 levels. **(A–C)** Representative IHC images showing *in situ* expression of GRP78 in the liver tissues. **(D)** Analysis of GRP78 protein immunohistochemical results. **(E–G)** Western blotting and qRT-PCR results showing GRP78 protein and mRNA levels. IOD, integrated optical density. ^∗∗^*P* < 0.01, ^∗^*P* < 0.05, vs. sham group; ##*P* < 0.01, vs. IRI group.

**FIGURE 6:**
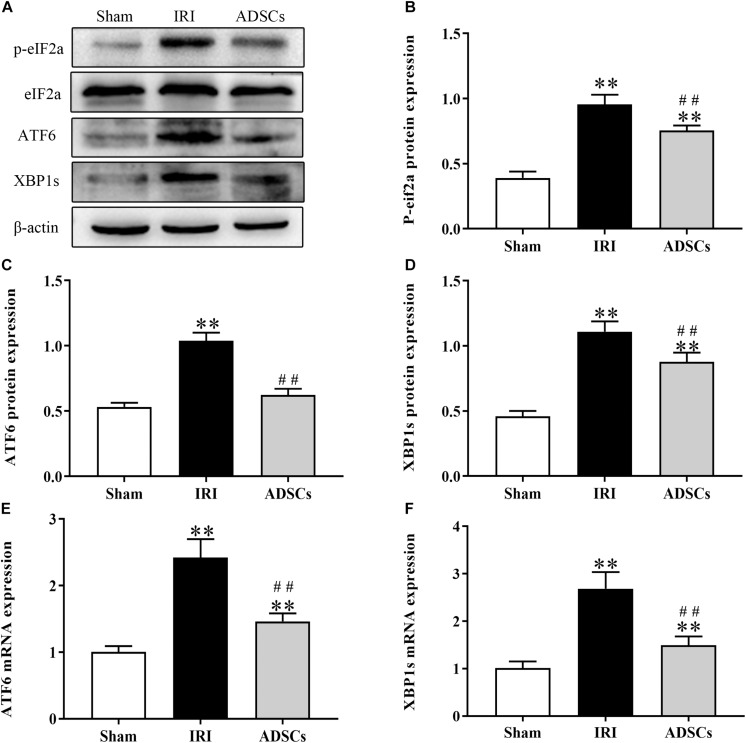
Effect of ADSCs transplantation on ERS-related protein levels. Western blotting and qRT-PCR results showing eIF2a, ATF6 and XBP1s protein **(A–D)** and mRNA **(E,F)** levels. ^∗∗^*P* < 0.01, vs. sham group; ##*P* < 0.01, vs. IRI group.

**FIGURE 7:**
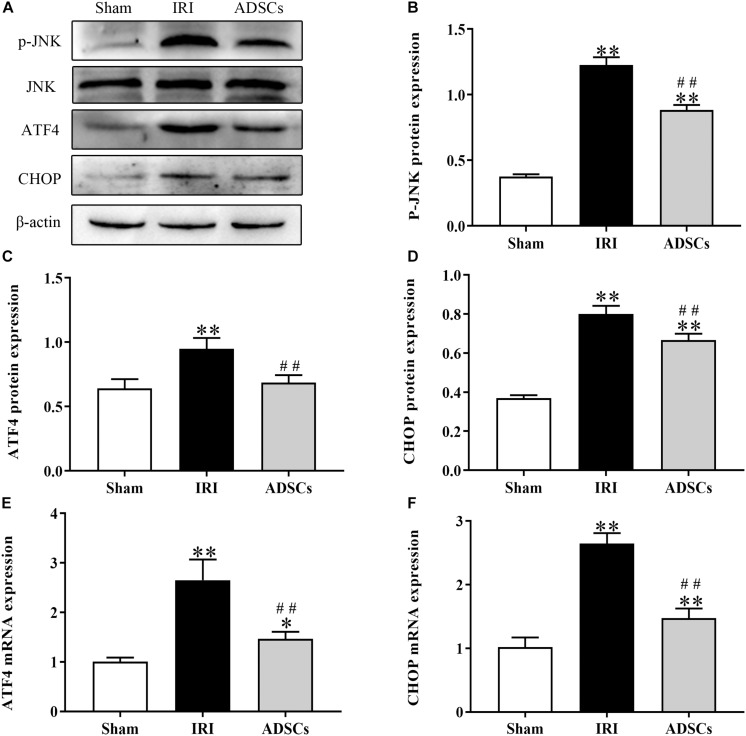
Effect of ADSCs transplantation on apoptosis-related proteins. Western blotting and qRT-PCR results showing JNK, ATF4 and CHOP protein **(A–D)** and mRNA **(E,F)** levels. ^∗∗^*P* < 0.01, ^∗^*P* < 0.05. vs. sham group; ##*P* < 0.01, vs. IRI group.

To determine whether ADSCs relieve ERS in the ischemic liver, we next analyzed the levels of ERS and apoptosis-related proteins in the different experimental groups. The ERS core protein GRP78 was markedly elevated in the hepatocyte cytoplasm following ischemia-reperfusion and partial hepatectomy compared to that in the sham-operated group. However, ADSC transplantation significantly downregulated GRP78 compared to the untreated animals 1 day after surgery (Figures 5A–F; *P* < 0.01 for all). Consistent with this, the GRP78 mRNA levels were also significantly higher in the IRI group relative to the sham-operated control (*P* < 0.05), and decreased in the ADSCs group at day 1 post-operation (Figure 5G; *P* < 0.01). The levels of the ERS-related proteins p-eIF2α, ATF6 and XBP1s were also significantly higher in the IRI compared to sham-operated and ADSCs-transplanted groups (*P* < 0.01). While ADSCs markedly decreased the level of ATF6 to baseline levels (*P* > 0.05 compared to sham-operated control), p-eIF2α and XBP1s expression levels were also reduced by the ADSCs (*P* < 0.01 compared to sham-operated control). Similar trends were seen with the transcripts of ERS-related genes (Figure 6). Since ERS is known to induce apoptosis, we also analyzed the levels of apoptosis-related proteins like p-JNK, ATF4 and CHOP in the liver tissues. As expected, IRI significantly increased the levels of the above factors (*P* < 0.01 compared to sham-operated control), all of which were downregulated by ADSCs (*P* < 0.01). Similarly, while both ATF4 and CHOP mRNAs were upregulated in the IRI group compared to the sham-operated and ADSCs groups (*P* < 0.01), and ADSCs downregulate their expression (Figure 7; *P* < 0.01 compared to IRI group). Taken together, ADSCs suppress ERS and ERs-induced apoptosis in the hepatocytes following hepatic IRI.

## Discussion

Recent studies have shown that ADSCs transplantation can attenuate liver IRI by reducing hepatocyte apoptosis (Ge et al., 2018a, b; Zhang et al., 2019). Furthermore, IR and partial hepatectomy injury triggers severe ERS via the PERK/eIF2α, IRE-1/XBP1 and ATF6 pathways, resulting in the accumulation of the ERS-related apoptosis protein CHOP. In agreement with previous findings, we observed that ADSCs ameliorated the pathological and microstructural changes in the liver parenchyma in a porcine model of hepatic IRI by alleviating hepatocyte ERS and apoptosis.

Adipose-derived mesenchymal stem cells are pluripotent stem cells that are abundant in the adipose tissue and can be easily isolated. In addition, they can rapidly expand *ex vivo* and maintain their pluripotency both *in vivo* and *in vitro*, making them ideal for cell-based regenerative therapy for various human diseases. Several studies have reported the therapeutic effects of mesenchymal stem cells (MSCs) isolated from the bone marrow and adipose tissues of mice (Tropel et al., 2004; Choi et al., 2014) and rats (Sgodda et al., 2007; Li et al., 2013), and confirmed their ability to differentiate into hepatocytes *in vitro* (Jiang et al., 2002; Winkler et al., 2015). Although translational research has been conducted on the hepatocyte differentiation of porcine MSCs (Groth et al., 2012), studies on ADSCs are still relatively few (Bosch et al., 2006). The Mesenchymal and Tissue Stem Cell Committee of the International Cell Therapy Association has proposed a minimum defined standard for characterizing human MSCs (Pittenger et al., 1999; Dominici et al., 2006), including adhesion to plastic, multi-directional differentiation ability and expression of distinct surface markers. The ADSCs extracted from the subcutaneous fat of pigs differentiate into osteogenic, adipogenic and hepatic cells, and express CD44, CD29, and CD90 but not CD34 (Brückner et al., 2013; Jiao et al., 2019). Based on these criteria, we successfully isolated and expanded porcine ADSCs *in vitro*.

The ER is a vital organelle involved in protein translation and post-transcriptional modification, calcium homeostasis and lipid biosynthesis (Avril et al., 2017; Zhang et al., 2017a). ER dysfunction leads to the accumulation of unfolded or misfolded proteins in its lumen, thereby triggering ERS via multiple pathways (Schröder and Kaufman, 2005). Studies increasingly show that ERS plays a major role in IRI of various organs (Thuerauf et al., 2006; Nakka et al., 2010). Liver IR leads to hypoxia, ATP/nutrient deprivation, oxidative stress and calcium overload (Liu et al., 2012), all of which can induce ERS (Chaudhari et al., 2014) that in turn triggers apoptotic cell death (Mizukami et al., 2010; Mosbah et al., 2010; Li et al., 2011). Since ERS is the immediate consequence of hepatocyte metabolic disorder, the pathological damage associated with IRI can be alleviated by inhibiting early ERS. Wang et al. showed that bone marrow-derived MSCs (BMSCs) inhibited ERS in the early stage of renal IR (Wang et al., 2019). Consistent with this, porcine ADSCs significantly alleviated the pathological damage and hepatocyte ERS induced by IR and partial hepatectomy injury.

The 78 KD glucose regulated protein (GRP78) is a molecular chaperone that is regulated by cellular glucose levels (Shiu and Pastan, 1979). The increase in the amount of misfolded or unfolded proteins during ERS leads to the decomposition of inactive GRP78 complexes and activation of the dissociated protein, which further aggravates ERS by initiating the requisite pathways (Hetz, 2012). This leads to an overall increase in GRP78 levels, increased synthesis of unfolded or misfolded proteins, and a net decrease in protein biosynthesis at the ER (Tabas, 2009; Guerrero-Hernandez et al., 2014). Therefore, altered expression level of GRP78 is an indicator of ER dysfunction and ERS. We observed an increase in the GRP78 protein and mRNA levels in the liver tissues after IRI, which was downregulated by the ADSCs. This clearly indicated that ERS was induced in the hepatocytes during IRI and alleviated by transplanting ADSCs.

The accumulation of unfolded or misfolded proteins in ER lumen further triggers the unfolded protein response (UPR) through RNA-dependent protein kinase (PKR)-like ER kinase (PERK), inositol-requiring enzyme 1α (IRE1α), and activating transcription factor 6 (ATF6). In the physiological state, these proteins are bound to GRP78 and thus inactive. When the ER homeostasis is disrupted, GRP78 dissociates from this complex and releases the other proteins as well. The subsequent increase in GRP78 levels and ATF6 activation are indicators of ERS (Guo et al., 2017). The ER membrane type I proteins PERK and IRE-1 are also activated by auto-dimerization and phosphorylation in the intracytoplasmic domain. PERK phosphorylates the eukaryotic initiation factor 2α (eIF2α) (Ghemrawi et al., 2018), and IRE-1 splices the 26 bp intron of the ATF6-induced XBP1 precursor mRNA resulting in the XBP1-spliced (XBP1s) transcript (Ron and Walter, 2007). Transcriptional activation of XBP1 is critical for UPR, which degrades ER-associated proteins and upregulates certain chaperone proteins (Lee et al., 2003). Liu et al. (2012) observed an increase in XBP1s and ATF6 levels during ERS early in liver reperfusion, which was also detected in our model of hepatic IRI. Chi et al. (2018) showed that ATF6, XBP-1 and p-eIF2α were significantly upregulated in rat brains subjected to middle cerebral artery occlusion (MCAO), and alleviated in the animals transplanted with ADSCs. In our study also, IRI significantly upregulated p-eIF2α and XBP1s, and promoted cleavage of full-length ATF6α. Thus, all three pathways of ERS were activated following hepatic ischemia-reperfusion. ADSCs down-regulated the above factors, indicating that their restorative effects were mediated by the inhibition of excessive ERS.

Although the primary function of UPR is to protect cells from stress-related damage, a sustained response in the event of severe ERS can also activate the JNK or caspase12-mediated apoptosis pathways to eliminate the damaged cells (Ding et al., 2014). Persistent ERS leads to the activation of ATF4 and XBP1s, resulting in the upregulation of the ERS-related apoptosis protein CHOP (Zhao et al., 2019). The PERK-eIF2α-ATF4 axis is necessary for CHOP expression (Fels and Koumenis, 2006), and its apoptotic effects are dependent on ATF4 (Li et al., 2009). Thus, activation of the PERK signaling pathway protects cells in the early stages of ERS by inhibiting protein synthesis. In the event of prolonged ERS, however, PERK promotes apoptosis via the eIF2α-ATF4-CHOP pathway. CHOP levels increased significantly in the brains of wild-type mice within 24 h of cerebral IR (Tajiri et al., 2004), further validating the relationship between IRI, prolonged ERS and apoptosis. The JNK activation pathway indirectly promotes ERS-induced apoptosis (Nieto-Miguel et al., 2007) via JNK1/2, which is known to regulate various pro-apoptotic and anti-apoptotic genes (Gorman et al., 2012). The apoptotic signaling molecule caspase 12 on the other hand has been detected in the ER of rodents but not in pigs (Szegezdi et al., 2006). In our study, CHOP and p-JNK were significantly elevated after IRI, indicating that both pathways are activated in the IR porcine liver due to excessive ERS. Consistent with the findings of Lee et al. (2019) that tonsil-derived MSCs have an inhibitory effect on CHOP, we found that the ADSCs also downregulated this protein in the injured liver. Thus, ADSCs can alleviate hepatic IRI damage by reducing ERS-induced apoptosis as well.

The current treatments in the pipeline for ERS and UPR rely on targeting ER calcium homeostasis, protein misfolding, Bip activators etc. (Ghemrawi et al., 2018). To the best of our knowledge, this is the first study to demonstrate that ADSCs can inhibit hepatic ERS following ischemic injury in a porcine model, an ideal animal model that shares considerable anatomical homology with humans. Although we did not track these cells *in vivo*, Ge et al. (2018b) detected fluorescently labeled ADSCs in the liver tissues of miniature pigs within 24 h of transplantation. In conclusion, ADSCs are a highly promising therapeutic tool against tissue injury and need further clinical validation.

## Conclusion

In conclusion, this study showed that ADSCs alleviated pathological damage and ultrastructural changes of hepatocytes following liver IRI by inhibiting ERS and reducing ERS-related hepatocyte apoptosis. Thus, these results providing new insights into cell-based therapies for hepatic IRI.

## Data Availability Statement

All datasets generated for this study are included in the article/supplementary material.

## Ethics Statement

The animal study was reviewed and approved by the Animal Care and Use Committee of the Northeast Agricultural University.

## Author Contributions

ZJ, XL, YM, QZ, BL, and HW performed the experiments. ZJ and HW conceived the study and designed the experiments. ZJ, XL, and YM collected the data. YG and QZ provided the technical guidance and interpreted the data. ZJ and BL analyzed the data and drafted the manuscript. All authors have read and approved the final manuscript.

## Conflict of Interest

The authors declare that the research was conducted in the absence of any commercial or financial relationships that could be construed as a potential conflict of interest.
